# Mitigating Oxidative Stress in Perinatal Cells: A Critical Step toward an Optimal Therapeutic Use in Regenerative Medicine

**DOI:** 10.3390/biom13060971

**Published:** 2023-06-10

**Authors:** Valeria Pizzuti, Francesca Paris, Pasquale Marrazzo, Laura Bonsi, Francesco Alviano

**Affiliations:** 1 Department of Medical and Surgical Sciences, University of Bologna, 40126 Bologna, Italy; valeria.pizzuti3@unibo.it (V.P.); francesca.paris6@unibo.it (F.P.); laura.bonsi@unibo.it (L.B.); 2 Department of Biomedical and Neuromotor Science, University of Bologna, 40126 Bologna, Italy; francesco.alviano@unibo.it

**Keywords:** oxidative stress, reactive oxygen species, cell senescence, antioxidants, mesenchymal stem cells, perinatal cells, regenerative medicine

## Abstract

Oxidative stress (OS) occurs when the production of reactive oxygen species (ROS) is not balanced by the body’s antioxidant defense system. OS can profoundly affect cellular health and function. ROS can have a profound negative impact on cells that undergo a predestined and time-regulated process of proliferation or differentiation, such as perinatal stem cells. Due to the large-scale employment of these immunotolerant stem cells in regenerative medicine, it is important to reduce OS to prevent them from losing function and increase their application in the regenerative medicine field. This goal can be achieved through a variety of strategies, such as the use of antioxidants and other compounds that can indirectly modulate the antioxidant defense system by enhancing cellular stress response pathways, including autophagy and mitochondrial function, thereby reducing ROS levels. This review aims to summarize information regarding OS mechanisms in perinatal stem cells and possible strategies for reducing their deleterious effects.

## 1. Oxidative Stress

Oxidative stress (OS) is a process in which the tissue levels of reactive oxygen species (ROS) rise above normal levels. This imbalance could be beneficial under physiological conditions but could also be harmful. At the physiological level, ROS play a crucial role in the immune system’s defense against pathogens and viruses. The release of ROS can also activate other immune cells and signaling pathways, leading to a robust immune response. Moreover, ROS have a fundamental role as redox messengers. Specifically, ROS regulate diverse biological processes, including gene expression, protein modification, and cell signaling. ROS-mediated signaling can involve the oxidation and reduction of specific amino acid residues, modulate enzyme activity, and impact the cellular redox state. On the other hand, OS can occur as a consequence of excessive ROS production due to external factors such as exposure to toxic substances, infection, radiation, pollution, and psychological stress. OS can indeed cause damage to many cellular components, including cellular membranes, proteins, lipids, and nucleic acids. ROS are produced during normal cellular metabolic activity. In recent years, OS has gained interest primarily because it has been investigated for its fundamental role in various clinical conditions, such as neurodegenerative and autoimmune diseases, viral and bacterial infections, and also for being largely responsible for cellular aging [1].

### 1.1. Reactive Oxygen Species Family

The term ROS encompasses various types of oxidant molecules, each with distinct properties and biological functions. It is fundamental to clarify the role of ROS, which can be beneficial or damaging depending on the circumstances [2]. The mechanisms and pathways involved in OS are multiple and complex. One of the main mechanisms involved is the formation of ROS from the electron transport systems in the mitochondria. These ROS can damage cellular components and cause an increase in oxidative damage, which can be further amplified by a dearth of antioxidants [2]. Within the family of ROS, the most medically relevant ones are superoxide anion, hydrogen peroxide, hydroxyl radical, singlet oxygen, and peroxynitrite.

The superoxide anion is a highly reactive free radical that is formed by the incomplete reduction of oxygen during the electron transport chain in mitochondria. It is also produced by other cellular enzymes, such as nicotinamide adenine dinucleotide phosphate (NADPH) oxidases. Superoxide can react with other molecules to form other ROS, such as hydrogen peroxide (H_2_O_2_). Superoxide anion is involved in various pathological processes, including inflammation, apoptosis, and aging, but notably, it has also been demonstrated by Muñoz et al. that superoxide anion can have an antioxidant role by chemically repairing DNA oxidized sites [3].

H_2_O_2_ is a stable ROS that is formed by the dismutation of the superoxide anion or by other cellular enzymes such as catalase and peroxidase. H_2_O_2_ can diffuse through membranes and can oxidize a variety of biomolecules, including proteins, lipids, and DNA. H_2_O_2_ plays an important role in bacterial and viral infections. Physiological production of H_2_O_2_, for example, by oral bacteria is a critical factor in maintaining equilibrium in tissue microecology due to its antimicrobial properties. In epithelial cells, the enzyme superoxide dismutase catalyzes the conversion of H_2_O_2_ to a superoxide ion, which plays important roles in cell signaling and host defense mechanisms [4].

Hydroxyl radical is the most reactive and damaging ROS; it can attack a wide range of biomolecules, including DNA, proteins, and lipids. Due to their high reactivity with various biomolecules, the diffusion distance of hydroxyl radicals in cells is believed to be very short [3,4]. Hydroxyl radicals are generated from H_2_O_2_ and superoxide anion in the presence of transition metal ions, such as iron or copper. Hydroxyl radicals are well known to be involved in various pathological processes, including cancer, neurodegenerative diseases, and aging.

Singlet oxygen is a highly reactive form of oxygen that is produced by the interactions between ultraviolet (UV) rays and molecules such as chlorophyll or porphyrins. Singlet oxygen can damage amino acids, DNA, and cellular membranes. In particular, the oxidation of fatty acids is a crucial mechanism since it induces the production of signaling molecules that are involved in carcinogenesis. In particular, singlet oxygen is involved in the pathogenesis of several skin diseases, including photoaging and skin cancer [5].

Peroxynitrite is a potent oxidant formed by the reaction of nitric oxide (NO) with superoxide anion. Peroxynitrite can damage various biomolecules, including proteins, lipids, and DNA, and is involved in the pathogenesis of several diseases, including neurodegenerative diseases, cardiovascular diseases, and cancer [6,7,8].

### 1.2. Oxidative Stress and Cellular Senescence

Cellular senescence is a condition in which cells are in a state of permanent cell proliferation and growth arrest even though they are still viable and metabolically active. Senescence cells usually display enlarged shapes, vacuoles, and cytoplasmic granules [9]. In addition to replicative senescence, which is characterized by a progressive reduction in telomere length in cells undergoing mitotic cell divisions and was first described by Hayflick and Moorehead [10], there are also a variety of senescence types that are related to extrinsic and/or intrinsic stress, for example, oncogene-induced senescence [11]. One major source of stress is OS induced by ROS [12,13]. In recent years, two theories associated with OS and senescence have been proposed. The first one considers the central role of mitochondria; failure in mitochondrial quality control systems, along with damage to mitochondrial genome integrity, is clearly associated with aging phenotypes [14]. The second and most recent hypothesis suggests that ROS may act as signaling molecules, mediating stress responses. In particular, the p53/p21WAF1 and Rb-p16INK4A molecular axes have been investigated in connection with cellular senescence since ROS may exacerbate senescence due to a positive feedback mechanism [13]. Moreover, ROS are also responsible for the acceleration of telomere loss because oxidative damage in the telomerase region is less efficiently repaired [15,16]. Cellular senescence also induces the secretion of senescence-associated secretory phenotype (SASP) factors. The SASP factors include many pro-oncogenic growth factors, proteases, and pro-inflammatory factors. Moreover, SASP factors promote ROS generation, itself leading to a reinforcing loop [17,18].

Stress-induced cellular senescence is a crucial aspect in cell therapy because it can causea reduction in cellular function and transplant efficiency. For this reason, it is important to deeply investigate the molecular aspects associated with cellular senescence and possible solutions to reduce or overcome this problem.

## 2. Regenerative Medicine and Perinatal Cells

Regenerative medicine is a promising field offering a wide range of potential treatments for various diseases and conditions, such as cardiovascular disease, diabetes, neurological disorders, orthopedic injuries, and cancer. One of the key principles adopted in regenerative medicine is the use of stem cells to replace damaged tissues. This is due to the stem cell’s ability to proliferate and differentiate. However, there are limitations associated with the use of stem cells, including the risk of tumor formation, immunoreactivity, and ethical controversies [19].

In recent years, there has been an increasing interest in finding new sources of cells that can be employed in regenerative medicine. Among the various cellular sources, perinatal cells have gained the most interest.

Perinatal cells are isolated from all the birth-associated tissues that are recovered from the term placenta and fetal annexes after delivery. Perinatal tissues comprise chorionic and amniotic membranes (including the epithelium), chorionic villi, the umbilical cord (including Wharton’s jelly), the basal plate (including maternal and fetal cells), and amniotic fluid [20]. Since placenta and fetal annexa are usually discarded after birth, they are considered waste products, for which reason the resulting cells isolated from such tissues are free of ethical implications, unlike other sources of stem cells. Moreover, one of the key advantages of perinatal cells is their ability to evade immune rejection when transplanted into a patient. Unlike other cell sources, such as adult stem cells, perinatal cells are immunologically immature, meaning that they do not express the same surface markers that trigger immune reactions. Due to this unique characteristic, perinatal cells display strong immunomodulatory properties [21,22] and can be transplanted into recipients without the need for immunosuppressive drugs, which may have serious side effects [20,22]. Perinatal cells have been found to interact with the immune system in a way that can be harnessed for therapeutic purposes. Immunomodulation occurs through a variety of mechanisms, including the release of soluble factors that inhibit the activation and proliferation of immune cells and the induction of regulatory immune cells that help maintain immune homeostasis. For example, perinatal cells have been shown to cause the differentiation of regulatory T cells, which may suppress the activity of effector T cells that drive inflammation and tissue damage [21].

Perinatal cells are also considered an attractive tool for regenerative medicine applications because of their remarkable ability to differentiate into various cell types, including bone, cartilage, muscle, and nerve cells [23,24].

This ability makes them a valuable tool for tissue engineering and regenerative medicine, as they can potentially be used to repair or replace damaged or diseased tissues.

Despite these advancements, regenerative medicine still faces challenges such as unwanted side effects resulting from the migration of transplanted cells and poor cell survival. Transplanted cells often have lower survival and proliferation rates due to a low blood supply and chronic inflammation present in the transplantation site, which can also impair cell activity in vivo [25].

Another significant issue in regenerative medicine is OS, which is associated with tissue injury and inflammation and can negatively impact various cellular processes such as cell adhesion, migration, and proliferation. Again, OS has been linked to cellular senescence, which can impair its regenerative and immunomodulatory activities [26]. Moreover, perinatal cells are particularly susceptible to OS in that they are in a state of rapid growth and development, demanding high levels of energy production and metabolism, which turn into a significant amount of ROS as a byproduct. In addition, perinatal cells are also exposed to OS during labor and delivery, which can lead to inflammation and further increase ROS production during cell culture. For this reason, it is essential to investigate the effect of OS on perinatal cells and study possible solutions to reduce the harmful effects and enhance the positive effects of perinatal cells in clinical applications.

## 3. Oxidative Stress and Perinatal Cells

The high energy demand by fetoplacental structures produces a physiological increase in the metabolic rate and mitochondrial activity, causing the generation of ROS and thus leading to an increase in the OS level and sterile inflammation. The OS-induced activation of the intrauterine inflammatory response is necessary to promote labor and delivery; these events are usually counterbalanced by the activation of antioxidant redox and anti-inflammatory systems. Dysfunction of these regulatory mechanisms causes an imbalance in the redox status, with consequences for placental structures and perinatal cell populations [27].

OS is a major cause of several pathological conditions related to pregnancy and delivery, including spontaneous preterm birth (PTB) and preterm prelabor rupture of the membranes (pPROM), with side effects also affecting fetal development and growth. It is a fact that the probability of pPROM and PTB onset increases after exposure to cigarette smoking, during infection, as well as in a condition of poor nutrition or obesity, which are all associated with dysregulation of the ROS balance.

Regarding the effect of ROS exposure on perinatal cells, amniotic epithelial cells (AEC) are known to be particularly exposed to OS; the studies by Menon and colleagues successfully described the impact of ROS-producing factors, such as water-soluble cigarette smoke extract (wsCSE), on AECs in vivo and during in vitro culture [28,29]. They showed histologic evidence of cellular senescence in patients with pPROM, describing alterations in nuclei, mitochondria, and the rough endoplasmic reticulum [28]. Menon and colleagues also reported that amniotic epithelial cells cultured in the presence of wsCSE showed activation of senescence-associated molecules such as SK1, P-p38 mitogen-activated protein kinase (MAPK), and p19, which promoted an increase in DNA damage and the onset of typical senescent features, including the release of SASP and Damage-Associated Molecular Pattern (DAMP) molecules and increased expression of Senescence-associated beta-galactosidase (SAβ-gal) [29]. Interestingly, the effect of wsCSE was partially prevented by exposure to the antioxidant N-acetyl-L-cysteine (NAC) [30]. It was also reported that AEC exosomes reflect the pathophysiologic features of cells exposed to CSE, showing an increase in H3, HSP70, and P-p38MAPK content, thus contributing to the release of senescence-associated signals in the surrounding environment [31].

Alterations to AEC characteristics during OS exposure are mainly related to hyperactivation of the p38MAPK signal, induced by both the apoptosis signal-regulating kinase (ASK1)-signalosome and transforming growth factor (TGF)-beta-activated kinase 1-binding (TAB1) protein 1 [32].

The increase in OS level may also be induced under in vitro culture conditions. Commonly used culture media are usually supplemented with serum or serum synthetic replacements and could be enriched with vitamins, amino acids, and heavy metal chelators, which are involved in ROS increase [33]. It has been observed that the addition of cysteine to chemically defined media markedly reduced the proliferation of Chinese hamster ovary cells seeded at low density, a fact related to an increase in OS [34]. Regarding the relevance of the culture medium, we also reported that during in vitro culture in traditional medium, AECs lose their native membrane lipid profile, with a decrease in polyunsaturated fatty acid (PUFA) content and particularly in omega 6. The changes in the AEC fatty acid profile caused the onset of senescent features, including an increase in cell dimension and the expression of senescence-associated genes such as p16 [35].

Similar results have also been obtained with fetal membrane mesenchymal stem cells (FM-MSCs). These perinatal cells, unlike AEC, are more prone to long-term in vitro culture; however, like the epithelial population, they show significant alteration of their membrane fatty acid profile between culture passages, with a loss of PUFA and an increase in monounsaturated fatty acid (MUFA) content compared to freshly isolated cells [36].

Mesenchymal stem cells (MSCs) from different sources have shown the capacity to migrate to the site of injury, where they can exert their anti-inflammatory and tolerogenic properties; however, the damaged tissue often releases high levels of inflammatory and OS mediators, which can alter the properties and phenotype of MSCs. Wharton’s jelly-derived MSCs (WJ-MSCs) represent one of the most promising MSC populations for cell therapy strategies. The effect of ROS was also assessed on the human WJ-MSC population, where Choo and colleagues reported that exposure of WJ-MSC to H_2_O_2_ caused growth arrest with the onset of premature senescence at early culture passages. As with other cell populations, senescent WJ-MSCs showed an increase in senescent SA β-Gal-positive cells and the expression of pro-apoptotic and senescence-associated genes [37]. The susceptibility of WJ-MSC to ROS is closely related to donor variability [38].

In the study conducted by Facchin and colleagues, the effect of H_2_O_2_ was evaluated on human WJ-MSCs at different culture passages, and the results were compared with those of H_2_O_2_-exposed adipose tissue-derived mesenchymal stem cells (AD-MSC) [39]. They observed that H_2_O_2_ exposure promoted the onset of the senescence-associated phenotype in both cell models, with a change in cell morphology along with a reduction in cell proliferation and cell viability, in a dose-dependent fashion. Interestingly, they investigated the different responses of WJ-MSCs and adult stem cells (ASCs) to OS exposure, highlighting the different proneness of MSC populations to undergoing senescence based on their tissue source.

The effect of OS caused by the in vitro culturing process has also been evaluated in Amniotic Fluid Mesenchymal Stem Cells (AF-MSCs). Compared with other MSC populations, these cells need to be cultured for many culture passages in order to obtain a significant cell number for potential therapeutic application, and this limitation increases their exposure to ROS [40].

**Figure 1:**
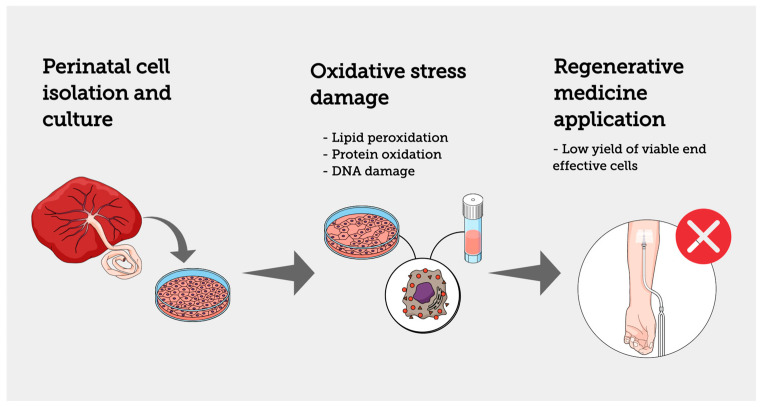
Perinatal cells are exposed to oxidative stress damage during in vitro culture and cryopreservation; the side effects of oxidative stress, such as lipid peroxidation, protein oxidation, and DNA damage, reduce the applicability of perinatal cells in regenerative medicine approaches.

The impact of OS has also been investigated on immortalized human amnion mesenchymal and epithelial cells. The results obtained indicated a different response to OS by the two amniotic cell populations: under normal conditions, amniotic membrane MSCs (AM-MSCs) expressed higher antioxidant factors, while when exposed to OS-inducing stimuli, the production of antioxidants was downregulated and AM-MSC apoptosis increased. In contrast, immortalized AECs expressed a higher level of basal antioxidant factors, which were upregulated under OS exposure, allowing cell survival [41]. The sensitivity of perinatal cells to ROS exposure may jeopardize the reliability of in vitro results, increasing the risk of misleading conclusions, so optimization of culture conditions and the use of antioxidant strategies are extremely necessary. Figure 1 summarize the side effects of oxidative stress on cultured or cryopreserved perinatal cells.

## 4. Antioxidants for Perinatal Cells

Antioxidant systems play a pivotal role in homeostasis and ROS regulation, acting via different pathways. The antioxidant capacity is based on three main mechanisms: hydrogen atom transfer, single electron transfer, and metal chelation. Some molecules possess direct antioxidant properties, while others exert their antioxidant effects indirectly.

Direct antioxidants can directly neutralize free radicals and oxidants through mechanisms such as electron donation. They act by directly scavenging ROS and interrupting radical oxidation reactions.

Indirect antioxidants, on the other hand, exert their antioxidant effects by activating cellular defense mechanisms or modulating signaling pathways. They may enhance endogenous antioxidant enzyme activity or promote the expression of cytoprotective genes. Compounds like sulforaphane, curcumin, and resveratrol fall into this category as they activate the Nrf2 pathway, leading to increased production of antioxidant enzymes and cytoprotective proteins.

Antioxidant molecules can act on different pathways, such as prevention of free radical formation, interruption of radical oxidation reactions, and inhibition of inactivated free radical/radical derivative reaction products. Superoxide dismutase (SOD), catalase (CAT), glutathione reductase (GR), and glutathione peroxidase (GPx) are widely known antioxidant enzymes, acting as first responders against ROS production since they convert reactive superoxide and H_2_O_2_ into water and oxygen [42]. Enzymes also act as a third-line defense through their detoxifying activity and ROS removal. Non-enzymatic antioxidants represent a second-line defense against ROS by rapidly inactivating radicals and oxidants. Several endogenous molecules, such as glutathione and coenzyme Q, exert essential antioxidant activities, while likewise exogenous substances obtained through diet, such as vitamins (A, C, and E), carotenoids, polyphenols, flavonoids, and bioflavonoids, are well known for their role in OS regulation [43,44]. NAC is a potent antioxidant that can be used to optimize in vitro conditions. As regards its action mechanisms, NAC acts as a precursor of reduced glutathione (GSH), a well-known direct antioxidant, and contributes to releasing free thiols and several reduced proteins with pronounced antioxidant activity [45,46].

Anthocyanins are molecules obtained from pigments found in fruits and vegetables and are known for their anti-inflammatory, antioxidant, and antidiabetic effects, among other properties [47].

Likewise, several herb-derived products have shown protective roles against damage caused by OS [48].

The transition from the in vivo environment to a culture system not suitable for mimicking the tissue of origin causes a serious imbalance between the levels of ROS and the antioxidant defenses activated by cells. Cell culture media are frequently deficient in important antioxidants that are normally obtained in the human diet, especially tocopherols, ascorbate, and selenium [49]. Compounds with antioxidant activity are optimal candidates for inclusion in cell culture protocols because of their safety and their ability to control OS.

Sovernigo and co-workers demonstrated that the addition of antioxidants during in vitro culture of bovine oocytes reduced ROS production and increased GSH levels. They also showed that supplementation of the culture medium with antioxidants promoted blastocyst development [50].

### 4.1. Antioxidants for Cryopreservation

Cryopreservation is a fundamental technique in regenerative medicine because it allows cells to be preserved for long periods at low temperatures, ensuring the survival of stem cells for transplantation. During cryopreservation, it has been demonstrated that OS may occur, resulting in damage to lipids, proteins, and DNA [51]. In order to improve the efficiency of cryopreservation, various antioxidant compounds have been studied as ways of increasing the resistance of cells to thermal stress [52]. Among these compounds, it has been demonstrated that NAC, thanks to its antioxidant power, is also capable of reducing the damage caused by cryopreservation [45,46]. Another natural compound, quercetin, has been proven not only to counteract the side effects related to cryopreservation but also to enhance the regenerative capacities of the human amniotic membrane, which is currently used in wound healing and burns [53]. Zang and colleagues have demonstrated that schisandrin B (SchB) supplemented in the storage solution preserved umbilical cord MSCs (UC-MSCs) from injuries associated with cryopreservation. The action mechanism of SchB seems to be associated with an antioxidant effect, mediated by nuclear factor erythroid 2-related factor 2 signaling, and an anti-apoptotic effect [54]. Antioxidant compounds have also been studied for preserving WJ-MSCs from cryopreservation. In particular, a combination of ROCK Y-27632 inhibitor, ascorbic acid, and trehalose has been supplemented in the culture medium and in the freezing medium, increasing viability and proliferation [55]. The effect of sulforaphane (SF) and epigallocatechin gallate (EGCG) cotreatment on thawed AF-MSCs has proven protective against stemness loss occurring after freeze-thaw cycles [36].

### 4.2. Antioxidant Supplementation for Stem Cell Culture

OS control is particularly important for the maintenance of stem cell properties during in vitro culture, which is an important prerequisite for their translation into cell therapy applications.

Several studies have shown that the ex vivo expansion of MSCs is associated with high genomic instability and reduced differentiation ability.

Several reports, obtained by studying several MSC sources, have provided evidence of antioxidant supplementation advantages for MSC cultures. For example, it has been observed that culturing isolated bone marrow MSCs (BM-MSCs) at a low O2 concentration (2%) or in the presence of NAC supplementation increases cell proliferation and genomic stability compared with normoxic conditions [56]. An increase in osteogenic and adipogenic differentiation ability was observed in BM-MSCs cultured in the presence of ascorbic acid 2-phosphate (AAP) [57]. Similar results were also observed in adipose-derived MSCs (AD-SCs) and in hematopoietic stem cells (HSCs) [58].

Since many perinatal cells display an MSC-like phenotype and promising therapeutic features, there are several studies based on antioxidant supplementation for the enhancement of stemness properties. In AF-MSCs, treatment with selenium and basic fibroblast growth factor (bFGF) reduced ROS accumulation and preserved MSC multipotency. Moreover, the conditioned medium of treated AF-MSCs has proven to be more effective in improving the proliferation and migration of fibroblasts [59]. AF-MSCs also showed an increased chondrogenic differentiation ability when cultured in the presence of sesamin [60]. Sesamin is known as an antioxidant compound involved in lipogenesis stimulation and the modulation of lipid composition [61].

Marrazzo and colleagues analyzed AF-MSCs obtained from healthy subjects exposed to OS factors, particularly in vitro-induced hyperoxia and freeze-thaw cycles. The presence in the culture medium of two antioxidants, such as SF and EGCG, increased the lifespan of AF-MSCs and reduced the relative percentage of senescent cells. Interestingly, the expression of stemness markers and antioxidant enzymatic effectors was enhanced, while a decrease in ROS production was observed [40]. Again, the combination of retinoic acid (RA), EGCG, and vitamin C with angiotensin II has been tested for improvement of cardiomyogenic differentiation in AF-MSCs [62]. Regarding the effect of antioxidant compounds on the senescence process that naturally occurs during in vitro culture, several compounds have been shown to counteract or delay the onset of senescence features in stem cells. Yuan and colleagues used Ganoderic Acid D (GA-D) during AM-MSC culture; they demonstrated that GA-D prevented OS-induced senescence by reducing SA-β-gal expression and enhancing the activity of antioxidant enzymes [63].

Several studies have reported the effect of resveratrol (RV) on the behavior of various stem cell populations. Prakoeswa and colleagues tested the addition of different doses of RV to WJ-MSC, AM-MSC, and AD-MSC. They concluded that RV enhanced the proliferation ability and the release of several growth factors in all the MSC populations, with a higher response observed in AD-MSCs [64].

As previously mentioned, the choice of culture medium also affects perinatal cell features since these cells are very sensitive to long-term in vitro maintenance. In a previous study, our group demonstrated that the addition of a tailored lipid supplement to the standard culture medium of FM-MSCs successfully restored a membrane fatty acid signature similar to the physiologic counterpart. The amelioration of the fatty acid profile improved the FM-MSC’s in vitro functional properties, including their proliferation rate, angiogenic differentiation, and immunomodulatory properties [36]. Similar results were obtained by using the tailored lipid supplement for the in vitro culture of the AEC population. The addition of a lipid supplement restored the AEC membrane fatty acid content, increasing the omega-6 percentage to physiological values. The recovery of an in vivo-like membrane fatty acid profile delayed the onset of senescence features and enhanced the immunomodulatory ability of AECs compared with standard culture conditions [35].

Hou and coworkers tested the effect of Vitamin C, a well-known natural antioxidant, on cultured AECs. Supplementation with Vitamin C significantly improved the cell’s life span and increased its proliferative and migratory activity. Treated AECs also displayed an increased expression of pluripotency markers. Finally, when transplanted into a mouse model of premature ovarian insufficiency, the treatment with Vitamin C enhanced the therapeutic potential of AECs, which contributed to rescuing ovarian function [65].

Vitamin C has also been combined with Vitamin E and Vitamin D3 as a pre-treatment cocktail for the culture of WJ-MSCs. From the results obtained, the antioxidant compounds increased survival among cells exposed to thermal stress and enhanced their migratory capacity and paracrine release [66].

The regulation of OS and ROS production is relevant for the maintenance of MSC features when it comes to applying them in cell therapy and regenerative medicine strategies [67]. It has been reported that MSCs derived from subjects with metabolic alterations, including metabolic syndrome and type 2 diabetes, are influenced by the high exposure to OS typical of these pathologic conditions. The surrounding environment impairs MSC abilities, and this could be a limitation for autologous transplantation [67,68].

Thus, the control of ROS exerted by intrinsic antioxidant systems or by external antioxidant supplementation contributes to the preservation of MSC properties.

**Figure 2:**
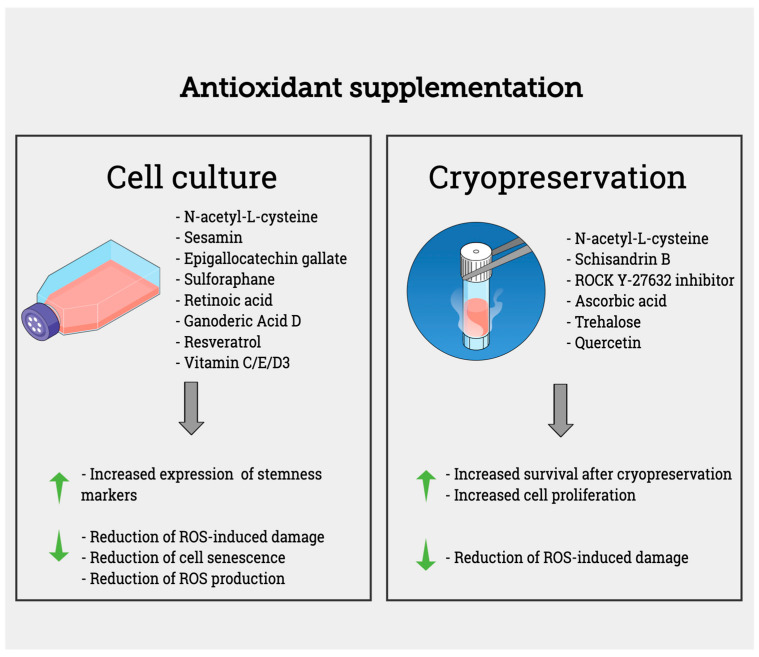
Antioxidant supplementation for in vitro culture and cryopreservation of stem cells. Supplementation of culture medium or freezing medium with antioxidant compounds contributes to the reduction of ROS-induced damage and senescence features while increasing stemness markers, cell survival, and proliferation.

In recent years, the application of MSCs themselves as potential intrinsic antioxidant tools has also been proposed and investigated. The antioxidant properties of MSCs are mainly related to their ability to increase endogenous antioxidant defenses and promote anti-inflammatory pathways [69]. Exosomes derived from UC-MSCs successfully reduced DNA oxidation and lipid peroxidation caused by cisplatin-induced kidney damage [70]. Similar results regarding the protective role of MSCs against ROS were obtained in animal models of diabetic retinopathy and nephropathy [71], in Alzheimer’s disease [72], and in mice models of premature aging [73]. The contribution of antioxidant compounds in reducing oxidative stress damages in cultured or cryopreserved stem cells is described in Figure 2.

## 5. Conclusions

The OS balance and ROS regulation are essential in many physiologic processes, including pregnancy, aging, and the immune response. Control of ROS production is particularly important in cell therapy approaches since stem cells, which are mainly involved in this therapeutic application, are particularly sensitive to ROS imbalance, resulting in functional impairment of cells reserved for clinical purposes. The imbalance in redox status caused by dysfunction of the regulatory mechanisms affects placental structures and various perinatal cell populations. Moreover, the exposure of perinatal cells to ROS, both in vivo and in vitro, activates senescence-associated molecules and results in DNA damage and the onset of the senescence phenotype. In this review, we have highlighted the effect of OS on perinatal cells since, due to their tissue of origin, these cells are highly exposed to ROS, which alters their characteristics, including during in vitro culture. Perinatal cells are widely studied for cell therapy application, so the development of antioxidant strategies, such as the use of proper culture media and supplementation with antioxidant compounds during in vitro culture, could be a useful approach to achieve while preserving functional cells for in vivo transplantation.

Further research is necessary to understand the molecular mechanisms involved in the effect of OS on perinatal cells. Nevertheless, the use of antioxidant supplements, such as the widely used NAC molecule, could be an effective strategy to prevent the onset of the senescence-associated phenotype in perinatal cells. Investigation of the impact of different culture media and specific supplements on perinatal cells could also lead to the development of novel culture conditions that better reconstitute the in vivo environment during in vitro culturing.

## Data Availability

Not applicable.

## Abbreviations

AAP	Ascorbic acid 2-phosphate
AD-MSC	Adipose tissue-derived mesenchymal stem cells
AD-SCs	Adipose-derived mesenchymal stem cells
AEC	Amniotic epithelial cells
AF-MSCs	Amniotic Fluid Mesenchymal Stem Cells
AM-MSCs	Amniotic membrane mesenchymal stem cells
ASCs	Adult stem cells
ASK1	Apoptosis signal-regulating kinase
bFGF	Fibroblast growth factor
BM-MSCs	Bone Marrow mesenchymal stem cells
CAT	Catalase
DAMPs	Damage-Associated Molecular Patterns
EGCG	Epigallocatechin gallate (EGCG)
FM-MSCs	Fetal membranes mesenchymal stem cells
GA-D	Ganoderic Acid D
GPx	Glutathione peroxidase
GR	Glutathione reductase
GSH	Reduced glutathione
H_2_O_2_	_H_ydrogen peroxide
HSCs	Hematopoietic stem cells
MAPK	Mitogen-activated protein kinase
MSCs	Mesenchymal stem cells
MUFA	Monounsaturated fatty acids
NAC	Antioxidant N-acetyl-L-cysteine
NADPH	Nicotinamide adenine dinucleotide phosphate
NO	Nitric oxide
OS	Oxidative stress
pPROM	Preterm prelabor rupture of the membranes
PTB	Preterm birth
PUFA	Polyunsaturated fatty acid
RA	Retinoic acid
ROS	Reactive oxygen species
RV	Resveratrol
SASP	Senescence-associated secretory phenotype
SAβ-gal	Senescence-associated beta-galactosidase
SchB	Schisandrin B
SF	Sulforaphane
SOD	Superoxide dismutase
TAB1	Activated kinase 1-binding
TGF	Transforming growth factor
UC-MSCs	Umbilical cord mesenchymal stem cells
UV	Ultraviolet
WJ-MSCs	Wharton’s jelly-derived mesenchymal stem cells
wsCSE	Water-soluble cigarette smoke extract
